# Mirror, Mirror on the Wall, How Does My Brain Recognize My Image at All?

**DOI:** 10.1371/journal.pone.0031452

**Published:** 2012-02-16

**Authors:** David L. Butler, Jason B. Mattingley, Ross Cunnington, Thomas Suddendorf

**Affiliations:** 1 School of Psychology, McElwain Building, University of Queensland, St. Lucia, Australia; 2 Queensland Brain Institute, QBI Building, University of Queensland, St. Lucia, Australia; Royal Holloway, University of London, United Kingdom

## Abstract

For decades researchers have used mirrors to study self-recognition. However, attempts to identify neural processes underlying this ability have used photographs instead. Here we used event related potentials (ERPs) to compare self-face recognition in photographs versus mirrors and found distinct neural signatures. Measures of visual self-recognition are therefore not independent of the medium employed.

## Introduction

Many people start their day with a look in the mirror. Yet despite interest from eminent scientists [1], [2], [3], [4], [5] it remains unclear how we recognize our own image (i.e. visual self-recognition). In a classic experiment, Gallup [4] found that chimpanzees were also capable of self-recognition, as they used mirrors to direct their behaviour towards an otherwise unseen novel mark placed upon their face. Subsequent studies have repeatedly shown that the only other primates that share this capacity are members of our closest living relatives, the great apes [4], [6], [7], [8], [9], [10], [11]. But not all humans recognize their own image. Children begin to develop self-recognition only between ages 18–24 months [12], [13]. In adults this ability can become diminished in conditions such as mirrored self-misidentification [14], body dysmorphic disorder [15], schizophrenia [16], and anorexia [17]. Recently, cognitive neuroscientists have attempted to identify the neural processes underlying this fundamental ability by studying participant's responses to images of their own faces (for reviews see [18], [19], [20], [21]). However, despite the widespread use of mirrors by both developmental and comparative psychologists (for reviews see [22], [23]), these studies have all used photographs rather than mirrors.

**Figure 1:**
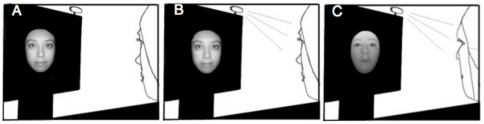
Black and white illustration of stimuli used. (A) Self photograph. (B) Self mirror. (C) Self wearing mask.

Can results involving photographs be generalised to mirrors and other media? A small number of developmental and neuropsychological findings suggest this may be problematic. For instance, children typically recognize themselves in mirrors before doing so in other media [24], [25], [26], [27]. In one study [24] using live, mirror reversed video images, children required an additional year before their passing rates were equivalent to self-recognition in mirrors. Up to 25% of Alzheimer's patients cannot recognize themselves in videos despite doing so in mirrors [28], and at least three cases have been reported showing the opposite pattern [14], [29], [30]. These apparent dissociations suggest that generalisations about the brain processes underlying self-recognition based solely upon studies using photographs may not be warranted. Here, for the first time, we examined neural activity in response to mirrors. We used Event Related Potentials (ERPs) to compare neural responses when seeing self in a mirror versus a photograph (see Figure 1).

## Results and Discussion

**Figure 2:**
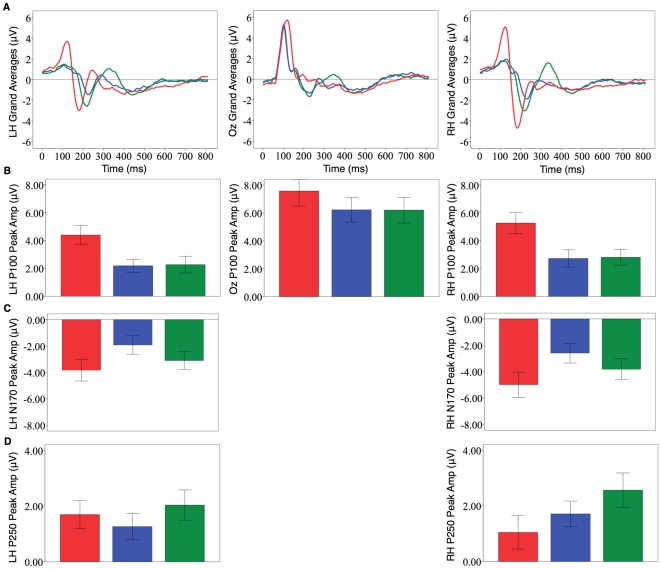
ERP activity. (A) Grand average ERPs for the P100, N170, and P250 (LH = Left Hemisphere, Oz = Midline, RH = Right Hemisphere; see Materials and Methods for details about which channels were selected) (self photograph = red, self mirror = blue, self wearing mask = green). (B) P100 peak amplitude. (C) N170 peak amplitude. (D) P250 peak amplitude. Differences emerged for all components when comparing self in mirrors vs photographs. Differences between self when unmasked and masked emerged for all ERP components except the P100.

Data are presented for three ERPs proposed to reflect three important stages of face processing [31], [32], [33], [34], [35], [36]. The grand averages and peak amplitudes for these ERPs are illustrated in Figure 2. An initial featural encoding stage occurs when the facial features are first detected (reflected by a positive peak of amplitude at around 100 ms; i.e. the P100). This is followed by a stage at which the configural relationship between features is analysed (reflected by a negative going peak at around 170 ms; i.e. the N170). A subsequent matching stage occurs when this newly constructed representation is compared to previously stored structural representations (reflected by a positive peak in amplitude at around 250 ms; i.e. P250).

Compared with mirror images, photographs of self produced a larger P100 amplitude with a longer latency (all reported findings use *p.*<.05 or Bonferroni adjustments; see Materials and Methods for full results). Furthermore, only photographs of self resulted in more P100 amplitude in the right compared to left hemisphere. For the N170, photographs produced more amplitude with an earlier latency. Finally, there were similar amplitudes and latencies for the P250, though differences between reflections and photographs emerged when considering cerebral hemisphere: only when viewing photographs was there more amplitude in the left compared to the right hemisphere. Together, these results show that self-recognition in different media involves distinct neural signatures in relation to the featural, configural, and matching stages of face recognition. These findings are consistent with developmental and neuropsychological research indicating that self-recognition may occur in one medium but not another [14], [24], [25], [26], [27], [29], [30].

Why does self-recognition in mirrors and photographs produce different neural signatures? Kinesthetic cues are available in mirrors but not in photographs, so this may potentially account for such differences. We think this is unlikely. Following standard ERP procedure, participants were asked to minimize movements as these produce artifacts that are removed from the data during analysis (see Materials and Methods). Furthermore, attention studies indicate that it takes at least 200 milliseconds to move one's eyes (let alone head) from fixation to a target (e.g., [37]), and our differences here are already being observed 100 milliseconds after the presentation of faces. Note also that kinesthetic cues may not be sufficient to pass mark tests [24], [25], [38]. For example, despite being able to observe their leg movement in a mirror, few children recognized their marked image when they were surreptitiously placed in novel pants. Children that had 30 seconds exposure to wearing these pants, on the other hand, passed the task [38].

Another potential factor is that photographs involve images of the past, while mirrors involve concurrent images in the here and now. Evidence supporting the possibility that temporal differences play a role comes from developmental studies where, despite recognizing one's image in live videos by 36 months, children still require an additional 12 months before showing equivalent passing rates for videos involving three-minute delays [24], [25], [27]. Photographs and reflections may also produce different affective responses. There is evidence for an affective processing route contributing to the recognition of familiar faces [39], and patients with dementia who can not recognize their own reflection may nonetheless experience strong emotional responses when presented with a mirror [40]. Finally, it is also possible that, given everyday experience with our reflections, we may have developed the expectation that when we look in a mirror we will see ourselves and not others. Such an expectation is unlikely to be that strong for photographs. We note that this explanation may also account for children's different performance between self-recognition in mirrors and videos [24]. Furthermore, it is more broadly consistent with the claim that expectations can alter brain processes underlying face recognition. For example, the amplitude of the N170 was found to change depending on whether participants knew the ambiguous stimuli they were looking at were faces or not [41]. Future research should examine what exactly causes these different neural responses to reflections and photographs of self.

The current study allowed us to address one more issue. Curiously, some individuals with mirrored self-misidentification can still recognize other people's reflections (e.g., [14]). This suggests that different neural processes may underlie the recognition of self and others in a mirror. It is exceedingly difficult to create a situation where mirror images of self and another person are equivalent in size, luminance, orientation, and location in space. We therefore asked participants to wear a facemask on some trials (see Figure 1). This allowed them to see two distinct facial features in a mirror under uniform conditions. We found no differences between reflections of self when unmasked or masked in the amplitudes or latencies of the P100, nor interactions with cerebral hemisphere for any ERP component. However, masked self produced larger amplitudes than unmasked self for both the N170 and P250. This suggests that when seen in a mirror, self and other faces result in similar featural encoding, but differences in configural analysis and matching. Though this is the first comparison of mirror images, similar differences in the N170 and P250 have been reported in ERP studies that compared photographs of self with photographs of unfamiliar faces (e.g., [31], [42]). It remains to be seen whether such differences also emerge when comparing self and familiar others in a mirror, as studies based upon photographs suggest these faces may differ in relation to the matching stage only (e.g., [31], [42], but see [43]).

This is the first study to examine neural responses to mirrors. The fact that we found distinct neural signatures of self-recognition in mirrors and photographs demonstrates that we cannot simply generalize findings from one medium to the other. Our paradigm raises the prospect of promising new avenues of inquiry that can shed light on vexing questions about how we recognize ourselves. Do ERPs change when young children first begin to recognize themselves in mirrors and again when they later come to recognize themselves in photographs and videos? How do ERPs in healthy people compare to those with conditions in which the capacity for self-recognition is distorted (e.g., anorexia) or impaired (e.g., mirrored self-misidentification)? To what extent are expectations about one's own appearance contributing to such conditions? Will humans and great apes share similar neural patterns for self-recognition using mirrors? The pursuit of such questions may go some way to unraveling the mysteries that have been raised by our obsession with that mirror on the wall.

## Materials and Methods

### Ethics Statement

Ethical clearance was granted from the University of Queensland's Ethics Committee (approval number: 08-PSYCH-PhD-42-CVH), which is in accordance with the regulations stipulated by the Australian National Health and Medical Research Council. Each participant gave informed written consent.

### Participants

Thirty-three people participated (13 males, 20 females), ranging from 24–39 years (*M* = 28.70 years, *SD* = 4.52). All were of Caucasian descent, had normal to corrected vision, and were right handed as determined by the Edinburgh Handedness Inventory [44]. Participation was rewarded with either course credit or payment (AUS$10.00 per hour).

### Stimuli and Materials

Stimuli consisted of faces that were presented either as a (1) mirrored reflection or (2) photograph. Participants viewed their mirrored reflection while either wearing a mask or no mask. The mask covered the entire face and was professionally coloured by a beauty therapist. Eye slits allowed participants to see out with minimal impairment. Photographs consisted of images of the participant and the mask (worn by the experimenter). Additional images were included to make the task more challenging and ensure participants were maintaining their attention. These were photographs of familiar (i.e. Justin Timberlake and Angelina Jolie) and unfamiliar faces. The inter-trial stimulus consisted of a grey and white checkerboard, the size of which matched the dimensions of the mirror.

Photographs of self were uniformly modified using Corell Paint Shop Pro (Corell Corporation, 2003) to be as similar as possible to the participant's reflection under experimental conditions (which was determined during pilot testing). This process involved self-photographs being: (1) mirror-reversed; (2) cropped at the chin, ears, and hairline (this was primarily determined by the outline of the head cover worn by the participant to cover up the electrodes); (3) adjusted in hue, luminance, and lighting (i.e., a lighting effect was used which gives the impression of lights shining down on the participant's face from above, as this occurred in the actual mirror conditions); (4) mounted onto a black background; (5) resized using a scale based upon the width of 250 pixels (this size was chosen because it equated with the visual angle of seeing one's reflection when sitting c. 90 cm from the mirror; although all participant's faces were rescaled to this width, the original ratio was maintained and this resulted in small differences in height between individuals); (6) converted into BMP format.

Located directly on top of the 30×40 cm screen of an NEC AccuSync computer monitor was a 17.5×12.5 cm double-sided mirror (Figures 1 and S1) This mirror remained on the screen over the same region where the photographs and inter-trial stimulus were presented. On the top right and left hand corners of the monitor were two Osram LED lights (wattage = 0.23; http://catalog.myosram.com). When these monitor lights were directed at the participant's face and the monitor screen behind the mirror was black, this allowed the participant to clearly see their own face in the mirror. When the monitor lights were turned off the mirror became transparent, allowing the participant to see images as they would normally be seen on the monitor screen.

The experimental task was designed and presented using E-prime software (www.pstnet.com/eprime). All instructions and images were displayed on a black background in the centre of the aforementioned monitor, with a resolution of 1024×768 pixels. Participant responses were recorded using a standard numerical keypad (arrow up = self; arrow right = familiar; arrow left = unfamiliar; and arrow down = mask). Response output was recorded by E-prime (for accuracy and reaction times) and Bio Semi (for EEG; http://www.bio-semi.com/).

### Experimental Task

There were six different types of block within the experiment, each of which consisted of trials predominantly coming from one of the six experimental conditions: self in photograph, familiar in photograph, unfamiliar in photograph, mask in photograph, self in mirror, and mask in mirror. A run occurred when each of these six blocks were presented without repeat. In total there were four runs (i.e., 24 blocks), the order of which was counterbalanced between participants (Table S1).

Each block consisted of pseudo-random trials numbering either 35 (for photographs) or 40 (for mirror images; this difference in trial number was due to the need for removing those mirror trials immediately following oddballs in the mirror blocks as these were likely to involve adjustments in eye accommodation-see below). Each face was shown for a maximum of 2000 ms, followed by the 1500 ms inter-trial stimulus. A response prior to 2000 ms would immediately result in the re-appearance of the inter-trial stimulus before going onto the next face. The total number of trials for each condition (excluding oddballs and accommodation trials) was 121 for photographs and 126 for mirror images.

For each block the participant would be predominantly presented with trials comprising of one particular face within a particular medium (e.g., self repeatedly seen in the mirror). Interspersed throughout these trials were also instances in which a non-predominant (i.e., oddball) face was presented (e.g., the unfamiliar photograph was seen in the predominantly self-mirror block; note that self and mask images were only ever presented in one medium within any given block, e.g., no trials of self in photograph were placed within a self in mirror block). We informed participants which face was going to be predominant at the start of *every* block given that turning the lights on already signalled that they would be most likely seeing mirrored reflections. However, we varied the number of oddballs that could be seen in any given block (between 1 and 9) to ensure that participants would actually attempt to identify the images rather than just blindly pressing the same button.

### Procedure

Participants were tested individually in a two-hour session in a dark room whilst sitting in a comfortable armchair. After application of the electrode cap, participants were fitted with a black cape, scarf, and head cover to ensure that only their face or the mask could be seen in the mirror. Participants were instructed to respond as quickly and accurately as possible to the identity of the face they saw as either self, mask, familiar or unfamiliar. At the beginning of each block participants were presented with information on the monitor indicating (1) which face would be most likely seen on the monitor or mirror during that block, (2) which buttons needed to be pressed for each face, and (3) which hand they had to use for their responses. Before the experiment started participants engaged in a practice session involving shortened blocks (i.e., 21 trials) for all conditions. During this practice, the experimenter asked participants to ensure that the mirror images were as similar as possible to the photographs in terms of size and luminance. This was accomplished by manipulating either the monitor lights and/or the participant's distance from the monitor. Following the experiment, most participants (starting from participant 10) were asked to indicate the degree of similarity between photographs and mirror images in terms of size and luminance (these ratings were: 5 = 0–5% variance, 4 = 10–15% variance, 3 = 20–30% variance, 2 = 30–40% variance, 1 = >40% variance; reported size rating: *M* = 4.09, *SE* = .09; reported luminance rating: *M* = 3.96, *SE* = .08).

### Electrophysiological Recording and Analyses

Event Related Potentials measure brain activity in the form of electrical amplitude as a function of time. Because millisecond resolution is attained, they afford the best opportunity to address the various stages involved in face recognition [14], [39], [45]. Electroencephalogram (EEG) data was continuously obtained using the Bio Semi Active Two system (http://www.bio-semi.com/) and analysed offline using BESA software (http://www.besa.de/index_home.htm). EEG was recorded using 64 Ag-AgCl electrodes fixed within an electrode cap according to the widening International 10–20 system [46] (Fp1, Fpz, Fp2, AF3, AF4, F7, F5, F3, F1, Fz, F2, F4, F6, F8, FT7, FC5, FC3, FC1, FCz, FC2, FC4, FC6, FT8, T7, C5, C3, C1, Cz, C2, C4, C6, T8, M1, TP7, CP5, CP3, CP1, CPz, CP2, CP4, CP6, TP8, M2, P7, P5, P3, P1, Pz, P2, P4, P6, P8, PO7, PO5, PO3, POz, PO4, PO6, PO8, CB1, O1, Oz, O2, CB2). The use of the Bio Semi Ag-AgCl active system reduces the need for skin preparation, and keeps impedance below 1Ω (see http://www.bio-semi.com/). To track eye movements we only recorded the horizontal electro-oculogram (EOG) by placing a pair of Ag-AgCl surface electrodes in a position where they could be covered by the black head cap (i.e., c. 2.5 cms laterally from the outer canthi of the left and right eyes). We did not record the vertical EOG as the placement of surface electrodes above and below an eye would be visible to the participant when looking at their mirrored reflection.

EEG and EOG signals were sampled at 1024 Hz with a band pass filter between 0.01–100 Hz. These signals were originally referenced to the CMS and DRL electrodes during data acquisition before being re-referenced offline to the average of the 64 channels (Bio Semi has replaced the need for ground channels with Common Mode Sense active channel, and Driven Right Leg passive electrode). Data were then segmented into 1250 ms epochs, with the 250 ms prior to stimulus onset used for the baseline correction. After blink artefact correction [47], EEG data were manually searched for EOG artefacts. BESA's artefact tool was then used for rejecting trials exceeding 100 µV. Oddballs, accommodation trials, and incorrect trials were excluded from analyses. EEG waveforms were then sorted with respect to condition and averaged to create ERPs for each participant. A minimal acceptance rate of 67 trials per condition was adopted, with most participants providing between 80 and 111 trials for each condition. ERPs were filtered with a high-pass filter of 0.1 Hz and a low-pass filter of 45 Hz (both with a slope of 12 dB/octave and of type zero phase). Grand average waveforms, averaged across all participants, were then calculated.

### Selection of Epochs and Channels for ERPs

Inspection of the grand average waveforms and topographical maps indicated the presence of the following sequence of components over posterior regions: a positive-going peak (P100), a negative-going peak (N170), and a second positive-going peak (P250). Peak amplitude was calculated as the measure for a component if the component was clearly defined relative to the baseline. The following components were subsequently measured as such: P100 (80–160 ms), N170 (140–270 ms), and P250 (200–400 ms). Channels were selected for each component where the peak amplitude was maximal. Over posterior regions, the channels used for each component were as follows: P100 (left hemisphere: P7, P9, PO7, O1; centre: Oz; right hemisphere: P8, P10, PO8, O2); N170 and P250 (left hemisphere: P7, P9, PO7; right hemisphere: P8, P10, PO8). We note that these epochs, channels, and regions are comparable to ones reported in prior self-recognition studies [43], [48], [49], [50], [51].

### Statistical Analyses

Accuracy rates for each condition were calculated as the percentage of correct responses relative to the total amount of correct and incorrect responses. Reaction times were also calculated as the amount of time (in milliseconds) between the presentation of the face and the participant's response to it. ERP data involved the amplitude and/or latency for each of the three main components discussed above: P100, N170, and P250. Because our primary concern was to address the possible effects of medium in self-recognition we first compared self in mirror and photographs. We predicted that self-recognition in photographs and mirrors would result in distinct ERPs for each component of face recognition. To test whether seeing one's own face in a mirror may be unique we then compared self in masked and unmasked mirror conditions. We predicted that differences between self when masked and unmasked would occur for the N170 and P250, but not the P100.

All analyses were performed using repeated measures ANOVA in SPSS (Version 17.0). Data were checked for normality using the Shapiro-Wilk test. When necessary, significant *p* values were adjusted using the Greenhouse-Geisser method for violations of sphericity, while the Bonferroni method was used for follow-up comparisons.

### Accuracy and Reaction Times

Participants were no more accurate when responding to self in photographs (*M* = 99.21%, *SE* = .23) than the mirror (*M* = 98.03%, *SE* = .79; *F*(1, 32) = 2.238, *p.* = .144; *η* ^2^ = .065). Furthermore, there was no difference between self when unmasked or masked (*M* = 97.73%, *SE* = 1.07; *F*(1, 32) = .060, *p*. = 808; *η* ^2^ = .002). No difference was found in reaction times between self in photographs (*M* = 481.61 ms, *SE = *10.78) and the mirror (*M* = 471.16 ms, *SE* = 12.45; *F*(1, 32) = 2.352, *p.* = .135; *η* ^2^ = .068), nor between self when unmasked or masked (*M* = 481.41 ms, *SE* = 11.98; *F*(1, 32) = 2.325, p. = .137; *η* ^2^ = .068).

### ERPs

**P100:** For medium, we found that photographs of self (*M* = 5.75 µV, *SE* = .38) produced a larger P100 compared to reflections of self (*M* = 3.72 µV, *SE* = .27; *F*(1, 32) = 68.233, *p.* = .000; *η* ^2^ = .681). A main effect for hemisphere (left: 3.29 µV, SE = .26; right: M = 4.00 µV, SE = .31; *F*(1.671, 53.473) = 71.802, *p*. = .000; *η* ^2^ = .692) was qualified by an interaction between medium and hemisphere (*F*(2, 64) = 6.910, *p*. = .002; *η* ^2^ = .178). Only when comparing photographs did the left hemisphere (*M* = 4.40 µV, *SE* = .35) show more amplitude than the right hemisphere (*M* = 5.28 µV, *SE* = .38, *t*(32) = −2.879, *p*. = .007; self mirror left: *M* = 2.19 µV, *SE* = .23; self mirror right: *M* = 2.73 µV, *SE* = .31; *t*(32) = −1.83, *p*. = .08).

For face identity we found no differences between reflections of self when unmasked or masked (*M* = 3.77 µV, *SE* = .30; *F*(1, 32) = .132, *p*. = .718; *η* ^2^ = .004). There was more amplitude in the right (*M* = 2.77, *SE* = .28) than left hemisphere (*M* = 2.23, *SE* = .26; *t*(32) = −2.046, *p*. = .049; *F*(2, 64) = 79.941, *p*. = .000; *η* ^2^ = .714). No interaction was found between face identity and hemisphere (self unmasked right: *M* = 2.73, *SE* = .31; self unmasked left: *M* = 2.19, *SE* = .23; self masked right: *M* = 2.82, *SE* = .28; self masked left: *M* = 2.27, *SE* = .30; *F*(1.644) = .293, *p*. = .704, *η* ^2^ = .009).

Severe violation of normality assumptions (Shapiro-Wilk <*p*. = .05) involving both mirror conditions lead to the removal of four outliers (remaining *N* = 28). Measuring latency at the channel where peak amplitude was highest (Oz), we found photographs of self (*M* = 117. 74 ms, *SE* = 3.09) resulted in a significantly later P100 compared to reflections of self (*M* = 101.15 ms, *SE* = 1.78; *F*(1, 27) = 25.978, *p*.<.000; *η* ^2^ = .490). For face identity there was no difference between self when unmasked or masked (*M* = 102.61 ms, *SE* = 1.70; *F*(1, 27) = 1.571, *p*. = .221; *η* ^2^ = .055).

**N170:** Violations of normality resulted in using data with two outliers being excluded (remaining *N* = 30). There was a larger negative amplitude for photographs of self (*M* = −4.41 µV, *SE* = .37) compared to reflections of self (*M* = −2.25 µV, *SE* = .33; (*F*(1, 29) = 68.705, *p.* = .000; *η* ^2^ = .703). The right hemisphere (*M* = −3.79 µV, *SE* = .40) had more negative amplitude than the left hemisphere (*M* = −2.87 µV, *SE* = .35; *F*(1, 29) = 6.232, *p.* = .018; *η* ^2^ = .177). No interaction was observed between medium and hemisphere (self photograph left: *M* = −3.82 µV, *SE* = .42; self photograph right: *M* = −4.99 µV, *SE* = .48; self mirror left: *M* = −1.92 µV, *SE* = .35; self mirror right: *M* = −2.59 µV, *SE* = .38; *F*(1, 29) = 1.760, *p*. = .195; *η* ^2^ = .057).

There was a larger negative amplitude for self masked (*M* = −3.46, *SE* = .30) than self unmasked (*F*(1, 29) = 46.247, *p*. = .000, *η* ^2^ = .615). There was a non-significant difference between left (*M* = −2.51, *SE* = .33) and right hemispheres (*M* = −3.20, *SE* = .37; *F*(1, 29) = 3.948, *p.* = .056; *η* ^2^ = .120). No interaction was found between face identity and hemisphere (self unmasked left: *M* = −1.92, *SE* = .35; self unmasked right: *M* = −2.59, *SE* = .38; self masked left: *M* = −3.10, *SE* = .34; self masked right: *M* = −3.82, SE = .40; *F*(1, 29) = .023, *p.* = .880; *η* ^2^ = .001).

The N170 occurred earlier for photographs of self (*M* = 192.42 ms, *SE* = 4.25) than reflections of self (*M* = 230.45 ms, *SE* = 3.66; (*F* (1, 32) = 70.761, *p.* = .000; *η* ^2^ = .689). For face identity, the N170 for masked self (*M* = 222.16 ms, *SE* = 4.06) occurred earlier than for unmasked self (*F*(1, 32) = 4.908, *p.* = .034; *η* ^2^ = .133).

**P250:** For medium, no difference in P250 amplitude was found between photographs of self (*M* = 1.37 µV, *SE* = .24) and reflections of self (*M* = 1.49 µV, *SE* = .21; *F*(1, 32) = .246, *p.* = .623; *η* ^2^ = .008). No difference was found between the hemispheres (left: *M* = 1.48 µV, *SE* = .20; right: *M* = 1.38 µV, *SE* = .22; (*F*(1, 32) = .365, *p.* = .550; *η* ^2^ = .011). An interaction between medium and hemisphere (*F*(1, 32) = 10.743, *p*. = .003; *η* ^2^ = .251) revealed no difference between left (*M* = 1.26, *SE* = .23) and right hemispheres (*M* = 1.71, *SE* = .23) for reflections of self (*t*(32) = −2.317, *p*. = .027), whilst for photographs of self there was more amplitude in the left (*M* = 1.70, *SE* = .25) compared to right hemisphere (*M* = 1.05, *SE* = .30; *t*(32) = 2.366, *p*. = .024).

For face identity self masked (*M* = 2.30, *SE* = .27) produced more amplitude than self unmasked (*F*(1, 32) = 9.558, *p*. = .004; *η* ^2^ = .230). The right hemisphere (*M* = 2.14, *SE* = .23) produced more amplitude than the left hemisphere (*M* = 1.65, *SE* = .19; *F*(1, 32) = 13.284, *p*. = .001; *η* ^2^ = .293). No interaction was observed between face identity and hemisphere (self unmasked left: *M* = 1.26 µV, *SE* = .23; self unmasked right: *M* = 1.71 µV, *SE* = .23; self masked left: *M* = 2.04 µV, *SE* = .27; self masked right: *M* = 2.57 µV, *SE* = .31; *F*(1, 32) = .057, *p*. = .813;*η* ^2^ = .002).

We observed no difference in P250 latency for photographs of self (*M* = 283.75 ms, *SE* = 7.95) compared to reflections of self (*M* = 290.16 ms, *SE* = 9.66; *F*(1, 32) = .392, *p.* = .536; *η* ^2^ = 0.12). For face identity, there was no difference in latency between self when unmasked or masked (*M* = 308.02 ms; *SE* = 8.30; *F*(1, 32) = 3.241, *p*. = .081; *η* ^2^ = .092).

## Supporting Information

Figure S1 **Diagram of experimental setup.**
(TIFF)

Table S1 **Outline of trials in each experimental condition/block.**
(DOC)
