# Supplementary figures and images for: Mirror, Mirror on the Wall, How Does My Brain Recognize My Image at All?
Source: PLoS One. 2012 Feb 16;7(2):e31452. doi: 10.1371/journal.pone.0031452 (PMC3281068; doi:10.1371/journal.pone.0031452)

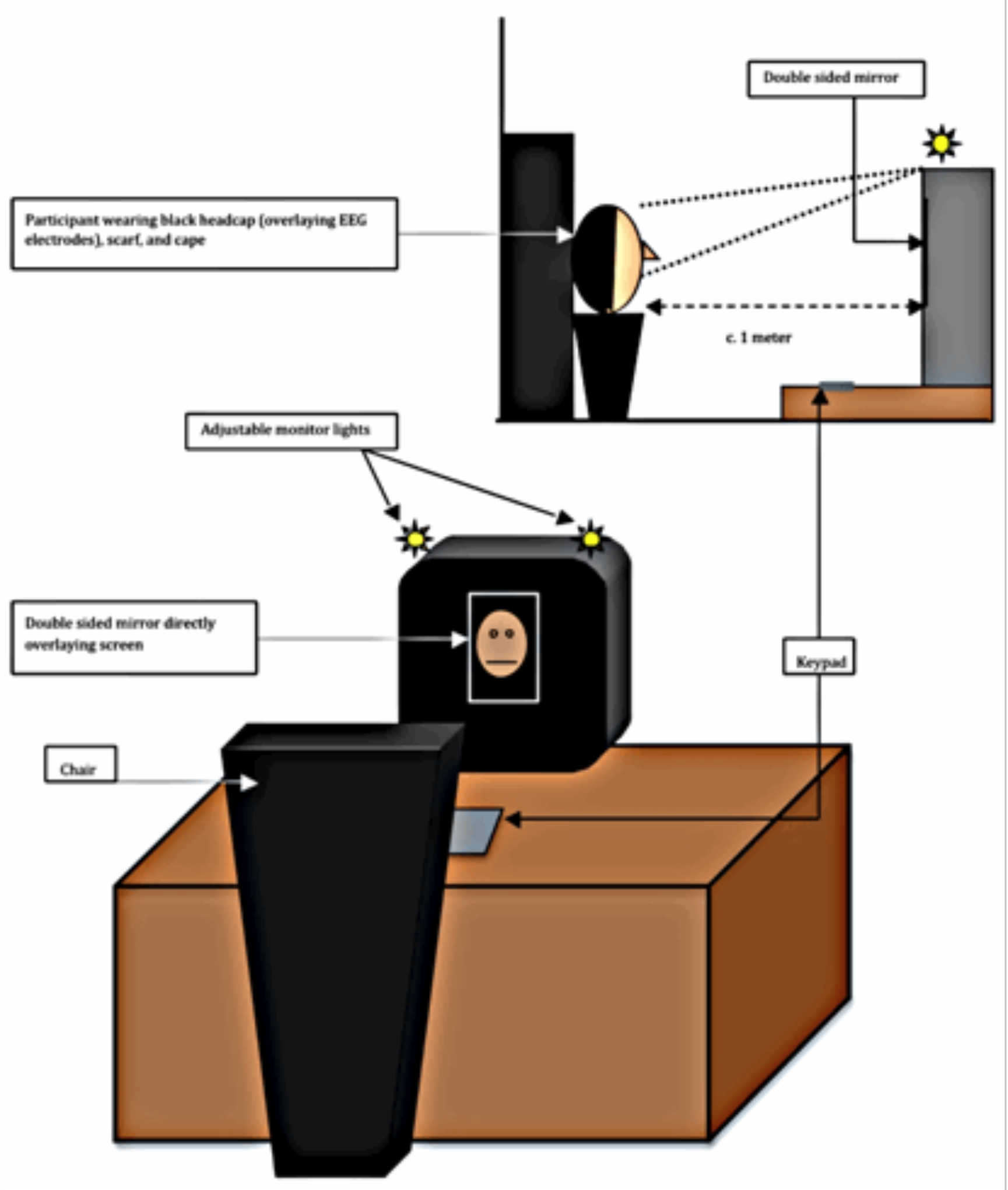

Supplement: Figure S1 — Diagram of experimental setup. (TIFF) [file pone.0031452.s001.tif]
