# Supplementary material for: Mirror, Mirror on the Wall, How Does My Brain Recognize My Image at All?
Source: PLoS One. 2012 Feb 16;7(2):e31452. doi: 10.1371/journal.pone.0031452 (PMC3281068; doi:10.1371/journal.pone.0031452)
Supplement: Table S1 — Outline of trials in each experimental condition/block. (DOC) [file pone.0031452.s002.doc]

|  | **Run 1** (left hand) | **Run 2** (right hand) | **Run 3** (left hand) | **Run 4** (right hand) |
| --- | --- | --- | --- | --- |
| Block 1 | Self (mirror) | Self (picture) | Mask (picture) | Unfamiliar (picture) |
| Trials | 34 self (6 others) | 32 self (3 others) | 29 mask (6 others) | 29 unfamiliar (6 others) |
| Block 2 | Familiar (picture) | Mask (picture) | Unfamiliar (picture) | Mask (mirror) |
| Trials | 26 familiar (9 others) | 26 mask (9 others) | 26 unfamiliar (9 others) | 37 mask (3 others) |
| Block 3 | Mask (mirror) | Mask (mirror) | Familiar (picture) | Self (mirror) |
| Trials | 39 mask (1 other) | 31 self (9 others) | 32 familiar (3 others) | 31 self (9 others) |
| Block 4 | Unfamiliar (picture) | Unfamiliar (picture) | Self (picture) | Mask (picture) |
| Trials | 32 unfamiliar (3 others) | 34 unfamiliar (1 other) | 34 self (1 other) | 32 mask (3 others) |
| Block 5 | Mask (picture) | Familiar (picture) | Self (mirror) | Self (picture) |
| Trials | 34 mask (1 other) | 29 familiar (6 others) | 39 self (1 other) | 29 self (6 others) |
| Block 6 | Self (picture) | Self (mirror) | Mask (mirror) | Familiar (picture) |
| Trials | 26 self (9 others) | 37 self (3 others) | 34 mask (6 others) | 34 familiar (1 other) |
